# Supplementary material for: Methodology to estimate natural- and vaccine-induced antibodies to SARS-CoV-2 in a large geographic region
Source: PLoS One. 2022 Sep 9;17(9):e0273694. doi: 10.1371/journal.pone.0273694 (PMC9462720; doi:10.1371/journal.pone.0273694)
Supplement: S1 Appendix — (PDF) [file pone.0273694.s001.pdf]

A Supplementary material to, "Estimation of total immunity to SARS-CoV-2 in Texas"

S.1 Order restricted (isotonic) estimation of probabilities

The algorithm below is retrieved from Algorithm 3 in `https://core.ac.uk/download/pdf/33107977.pdf`. The maximum likelihood estimate of $\pi = (\pi_1, \pi_2, \ldots, \pi_H)$ under the restriction of $\pi_1 \leq \pi_2 \leq \cdots \leq \pi_H$, is calculated in the following way: Let n_h the number of observations in group h,

Step 1: Do $\tilde{\pi}_h$ equal to the sample proportion in group h.

Step 2: While not $\tilde{\pi}_h \leq \tilde{\pi}_{h+1}$, for $h = 1, \ldots, H-1$, do

For $h = 1, \ldots, H$

If $\tilde{\pi}_h \not\leq \tilde{\pi}_{h+1}$ do

$$\tilde{\pi}_h = \frac{n_h}{n_h + n_{h+1}} \tilde{\pi}_h + \frac{n_{h+1}}{n_h + n_{h+1}} \tilde{\pi}_{h+1}, \quad \text{and} \quad \tilde{\pi}_{h+1} = \tilde{\pi}_h.$$

S.2 Confidence interval for the seroprevalence

The construction of the confidence interval for $\widetilde{SPR}_t$ is based on the confidence interval for a proportion under a stratified sampling design [26, 27]. Recall, the weight $w_h = N_h/N$ and N_h denote the proportion of and the number of individuals in the population in the age group h, respectively, and N the population total. An estimate of the sampling variance of the sample proportion $\dot{\eta}_{ht}$ is

$$\widetilde{\text{Var}}(\dot{\eta}_{ht}) = \left[\frac{N_h - n_{ht}}{N_h - 1}\right] \frac{\dot{\eta}_{ht}(1 - \dot{\eta}_{ht})}{n_{ht}} \approx \frac{\dot{\eta}_{ht}(1 - \dot{\eta}_{ht})}{n_{ht}}$$

where n_{ht} is the number of participants in age group h (at week t). The approximation in the above equation is valid since $N_h >> n_{ht}$. We base the confidence interval on this equation plugging in $\tilde{\eta}_{ht}$ instead of $\dot{\eta}_{ht}$

$$\widetilde{\text{Var}}(\tilde{\eta}_{ht}) \approx \frac{\tilde{\eta}_{ht}(1 - \tilde{\eta}_{ht})}{n_{ht}}$$

Then the sampling variance of $\widetilde{SPR}_t$ is estimated with

$$\widetilde{\text{Var}}(\widetilde{SPR}_t) = \sum_h w_h^2 \widetilde{\text{Var}}(\tilde{\eta}_{ht}) \approx \sum_h w_h^2 \frac{\tilde{\eta}_{ht}(1 - \tilde{\eta}_{ht})}{n_{ht}}$$

The asymptotic $1 - \alpha$ confidence interval for SPR_t is then

$$\widetilde{SPR}_t \pm z_{1-\alpha/2} \sqrt{\widetilde{\text{Var}}(\widetilde{SPR}_t)}$$

where z_α is the percentile α of the standard normal distribution, so when $1 - \alpha = 0.95$, $z_{1-\alpha/2} = 1.96$.
