# Supplementary material for: Methodology to estimate natural- and vaccine-induced antibodies to SARS-CoV-2 in a large geographic region
Source: PLoS One. 2022 Sep 9;17(9):e0273694. doi: 10.1371/journal.pone.0273694 (PMC9462720; doi:10.1371/journal.pone.0273694)
Supplement: Attachment — Submitted filename: PLOSONEresponsetoreviewers.docx [file pone.0273694.s002.docx]

Luis Novelo-Leon

1200 Pressler St Suite 920

Houston, Texas 77030

July 27, 2021

Dear Dr Janin,

Thank you greatly for a favorable review of our manuscript, and the support to resubmit.

Please see our point-by-point response/rebuttal letter here (in red), our uploaded marked copy, and the unmarked version of our revised manuscript. We thank the reviewers for their excellent points and the ability to clarify the paper. We have incorporated many of their edits for clarity, including updating Table 1 to show period seropositivity of our cohort by demographics, and updating explanations and figures per reviewer suggestions.

**From the Associate Editor/Editorial Board:**

When submitting your revision, we need you to address these additional requirements.

1. Please ensure that your manuscript meets PLOS ONE's style requirements, including those for file naming. The PLOS ONE style templates can be found at

<https://journals.plos.org/plosone/s/file?id=wjVg/PLOSOne_formatting_sample_main_body.pdf>and

[https://journals.plos.org/plosone/s/file?id=ba62/PLOSOne_formatting_sample_title_authors_affiliations.pdf](https://urldefense.proofpoint.com/v2/url?u=https-3A__journals.plos.org_plosone_s_file-3Fid-3Dba62_PLOSOne-5Fformatting-5Fsample-5Ftitle-5Fauthors-5Faffiliations.pdf&d=DwMGaQ&c=bKRySV-ouEg_AT-w2QWsTdd9X__KYh9Eq2fdmQDVZgw&r=G4zWUYoqEhZntfJlQG-0ibiHLmNKNGYaYv3iNo-LeeY&m=H1kaiBZAC16dyMBoyhrZah25GXw4hdDRBm7TMwv6gRM&s=GMcC3qmG3P4OX8rlXubMt_Rk8Vib4e56UMEtinhk4P0&e=).

We have now used PLOS latex template for this submission!

2.  Please amend your current ethics statement to address the following concerns:

a) Did participants provide their written or verbal informed consent to participate in this study?

b) If consent was verbal, please explain i) why written consent was not obtained, ii) how you documented participant consent, and iii) whether the ethics committees/IRB approved this consent procedure.

Thank you. Regarding 2 a,b, this has been added to the Methods Section, “All protocols were reviewed and approved by the University of Texas Health Science Center’s Committee for the Protection of Human Subjects, and were also deemed public health practice by the Texas Department of State Health Services IRB. All adults consented electronically to be in the study, and children under 18 either consented or assented to be in the study.”

3. Please update your submission to use the PLOS LaTeX template. The template and more information on our requirements for LaTeX submissions can be found at [http://journals.plos.org/plosone/s/latex](https://urldefense.proofpoint.com/v2/url?u=http-3A__journals.plos.org_plosone_s_latex&d=DwMGaQ&c=bKRySV-ouEg_AT-w2QWsTdd9X__KYh9Eq2fdmQDVZgw&r=G4zWUYoqEhZntfJlQG-0ibiHLmNKNGYaYv3iNo-LeeY&m=H1kaiBZAC16dyMBoyhrZah25GXw4hdDRBm7TMwv6gRM&s=L2HPUpOOVrHl8kU6y5LjjTfHd4pJNxEIuyOMcg85BxY&e=).

This has been done.

4. Thank you for stating the following financial disclosure:

“Project funded by Texas Department of State Health Services (Grant #HHS000866600001)”

Please state what role the funders took in the study.  If the funders had no role, please state: ""The funders had no role in study design, data collection and analysis, decision to publish, or preparation of the manuscript.""

If this statement is not correct you must amend it as needed.

Thank you, the award statement is correct.

Please include this amended Role of Funder statement in your cover letter; we will change the online submission form on your behalf.

We would like to amend Role of Funder to,

“Funding partners met/meet weekly with the state funding team to review survey findings and two representatives of the funding team are also co-authors on this manuscript. “

5. We note that the grant information you provided in the ‘Funding Information’ and ‘Financial Disclosure’ sections do not match. When you resubmit, please ensure that you provide the correct grant numbers for the awards you received for your study in the ‘Funding Information’ section.

We have removed the grant # from the manuscript as requested in Editor’s comments 5-6.

For our Financial Disclosure form you may write, “The study was funded by Texas Department of State Health Services (Grant #HHS000866600001).”

6. Thank you for stating the following in the Funding Section of your manuscript:

“Project funded by Texas Department of State Health Services (Grant #HHS000866600001)”

We note that you have provided funding information that is not currently declared in your Funding Statement. However, funding information should not appear in the Acknowledgments section or other areas of your manuscript. We will only publish funding information present in the Funding Statement section of the online submission form.

Please remove any funding-related text from the manuscript and let us know how you would like to update your Funding Statement. Currently, your Funding Statement reads as follows:

“Project funded by Texas Department of State Health Services (Grant #HHS000866600001)”

This funding number and institution above are correct. And this has now been removed from the manuscript itself.

Please include your amended statements within your cover letter; we will change the online submission form on your behalf.

7. In your Data Availability statement, you have not specified where the minimal data set underlying the results described in your manuscript can be found. PLOS defines a study's minimal data set as the underlying data used to reach the conclusions drawn in the manuscript and any additional data required to replicate the reported study findings in their entirety. All PLOS journals require that the minimal data set be made fully available. For more information about our data policy, please see <http://journals.plos.org/plosone/s/data-availability>.

Upon re-submitting your revised manuscript, please upload your study’s minimal underlying data set as either Supporting Information files or to a stable, public repository and include the relevant URLs, DOIs, or accession numbers within your revised cover letter. For a list of acceptable repositories, please see <http://journals.plos.org/plosone/s/data-availability#loc-recommended-repositories>. Any potentially identifying patient information must be fully anonymized.

Important: If there are ethical or legal restrictions to sharing your data publicly, please explain these restrictions in detail. Please see our guidelines for more information on what we consider unacceptable restrictions to publicly sharing data: <http://journals.plos.org/plosone/s/data-availability#loc-unacceptable-data-access-restrictions>. Note that it is not acceptable for the authors to be the sole named individuals responsible for ensuring data access.

We will update your Data Availability statement to reflect the information you provide in your cover letter.

The funders currently do not allow sharing of individual level data; however, the TX CARES team shares granular data on a weekly updated public Dashboard. Below is the “Data Sharing Statement” required by our funders, which we have now added to the manuscript Methods section as well as the Dashboard link below. Here is their data sharing agreement,

**Data Sharing Statement:** “Texas CARES investigators are committed to data sharing. Granular results and user-specified data summaries are currently publicly available on the Texas CARES portal (<https://sph.uth.edu/projects/texascares/dashboard>). When baseline recruitment is complete, a de-identified individual level dataset will be available for download from the same portal.”

| <https://sph.uth.edu/projects/texascares/dashboard> | [TXCARES DASHBOARD - TexasCARES - The University of Texas Health Science Center at Houston (UTHealth) School of Public Health](https://sph.uth.edu/projects/texascares/dashboard)  Home to diverse communities that reflect the future population of the US, students at the UTHealth School of Public Health in Houston are uniquely positioned to gain hands-on experience on local projects with national significance. Our faculty are pioneering radical solutions for imminent public health problems and provide the tools and resources that will push you to think critically and ...  sph.uth.edu |
| --- | --- |

8. We note that you have indicated that data from this study are available upon request. PLOS only allows data to be available upon request if there are legal or ethical restrictions on sharing data publicly. For more information on unacceptable data access restrictions, please see <http://journals.plos.org/plosone/s/data-availability#loc-unacceptable-data-access-restrictions>.

In your revised cover letter, please address the following prompts:

a) If there are ethical or legal restrictions on sharing a de-identified data set, please explain them in detail (e.g., data contain potentially sensitive information, data are owned by a third-party organization, etc.) and who has imposed them (e.g., an ethics committee). Please also provide contact information for a data access committee, ethics committee, or other institutional body to which data requests may be sent.

As stated above, the funders (Department of State Health Services, DSHS of Texas) own the data. The DSHS will make the decision to release individual level de-identified data when baseline recruitment is complete. Until then, granular data are continually reported per the above Dashboard, which is now referenced in the paper as well.

b) If there are no restrictions, please upload the minimal anonymized data set necessary to replicate your study findings as either Supporting Information files or to a stable, public repository and provide us with the relevant URLs, DOIs, or accession numbers. For a list of acceptable repositories, please see <http://journals.plos.org/plosone/s/data-availability#loc-recommended-repositories>.

We will update your Data Availability statement on your behalf to reflect the information you provide.

Please see above response to a.)

**RESPONSES TO REVIEWERS 1 & 2**

Reviewer #1: This study describes a statistical method to combine serial anti-nucleoprotein antibody testing and vaccination data to estimate the prevalence of SARS-CoV-2 antibodies in Texas due to natural infection, vaccination and both. The estimates given are for the overall state, but it would be more valuable to have these at finer resolution, e.g. by age groups, gender, ethnicity, county level, etc. if the data is available.

First, we note that we have updated Table 1 to include seropositivity (i.e., period seroprevalence) for a 30-day period, by all demographics. This 30-day period was the most contemporary and most relevant.

For additional granular demographic and county data, we refer the reviewer to the publicly available Dashboard that displays weekly seroprevalence for natural infection by all demographics, populations studied, counties, etc. However, we do not have this information for vaccinated individuals in TX as these data are owned by the state of Texas and are not shared at this granular level, but the state vaccination Dashboard is now referenced in our paper, along with the TX CARES Dashboard that shows country maps,

(<https://sph.uth.edu/projects/texascares/dashboard>).

Methods:
State whether the Texas CARES population is from all over Texas or just confined to certain cities/counties.

Thank you for this important question. We now add the following text to our Methods section and also refer the reviewer to the dashboard “enrollment map” on the right-hand side of the below link (also pasted below this response).

“The catchment area for the cohort study was the state of Texas. Recruitment efforts were taken to enroll rural and urban participants spanning the state.” This was accomplished by liaising with FQHCs in geographically diverse areas, holding blood draw events in rural and urban areas, distributing information, flyers, and mailings to health clinics and universities throughout the state. In the time this paper was under revision, we also published the study design in Valerio-Shewmaker, 2021, and refer readers to that paper and the others that were published in this time frame:

1. Valerio-Shewmaker MA, DeSantis SM, Swartz MD, Yaseen A, Gonzalez MO, Kohl HB III, Kelder SH, Messiah SE, Aguillard KA, Breaux C, Wu L, Shuford J, Pont S, Lakey D, Boerwinkle E. (2021). Strategies to Estimate Prevalence of SARS-CoV-2 Antibodies in a Texas Vulnerable Population: Results from Phase I of the Texas Coronavirus Antibody REsponse Survey (TX CARES), *Frontiers in Public Health* Dec 14;9:753487.
2. Messiah SE, Hao T, DeSantis SM et al (2022). Comparison of persistent symptoms of SARS-CoV-2 infection by antibody status in non-hospitalized children and adolescents. *The Pediatric Infectious Disease Journal*.
3. Messiah SE, DeSantis S, Leon-Novelo L, Talebi Y, Brito F, Kohl III HW, Valerio-Shewmaker M, Ross J, Swartz MD, Yaseen A, Kelder SH, #Zhang S, Omega-Njemnobi OS, Gonzalez MO, Wu L, Boerwinkle E, Lakey D, Shuford JA, Pont SJ. Durability of SARS-CoV-2 Antibodies From Natural Infection in Children and Adolescents. *Pediatrics*. 2022 Mar 18. doi: 10.1542/peds.2021-055505. Epub ahead of print. PMID: 35301530.
4. Swartz MD, DeSantis SM, Yaseen A, et al. (20220). Antibody duration after infection from SARS-CoV-2 in the Texas Coronavirus Antibody Response Survey. Accepted to *Journal of Infectious Diseases*.

Our Dashboard is here:

<https://sph.uth.edu/projects/texascares/dashboard> (please click on “enrollment map” to see the below)

Below displays a heatmap of total TX CARES enrollment (e.g. blood draw), by county to demonstrate representation of Texas as of Oct 31, 2021. A version of this map is linked to in the manuscript (our public Dashboard). 229 Texas counties are represented at the time of this submission. They are in list form next to the heatmap.

| No | County | Count | Percentage |
| --- | --- | --- | --- |
| 1 | ANDERSON | 136 | 0.15 % |
| 2 | ANDREWS | 4 | 0 % |
| 3 | ANGELINA | 435 | 0.49 % |
| 4 | ARANSAS | 128 | 0.14 % |
| 5 | Archer | 134 | 0.15 % |
| 6 | ARMSTRONG | 5 | 0.01 % |
| 7 | ATASCOSA | 58 | 0.07 % |
| 8 | AUSTIN | 41 | 0.05 % |
| 9 | BANDERA | 107 | 0.12 % |
| 10 | BASTROP | 423 | 0.48 % |
| 11 | BAYLOR | 4 | 0 % |
| 12 | BEE | 17 | 0.02 % |
| 13 | BELL | 659 | 0.75 % |
| 14 | BEXAR | 6791 | 7.68 % |
| 15 | BLANCO | 86 | 0.1 % |
| 16 | BOSQUE | 39 | 0.04 % |
| 17 | BOWIE | 86 | 0.1 % |
| 18 | BRAZORIA | 698 | 0.79 % |
| 19 | BRAZOS | 1107 | 1.25 % |
| 20 | BREWSTER | 5 | 0.01 % |
| 21 | BROOKS | 3 | 0 % |
| 22 | BROWN | 16 | 0.02 % |
| 23 | BURLESON | 71 | 0.08 % |
| 24 | BURNET | 419 | 0.47 % |
| 25 | CALDWELL | 190 | 0.21 % |
| 26 | CALHOUN | 67 | 0.08 % |
| 27 | CALLAHAN | 34 | 0.04 % |
| 28 | CAMERON | 501 | 0.57 % |
| 29 | Camp | 10 | 0.01 % |
| 30 | CARSON | 1 | 0 % |
| 31 | CASS | 20 | 0.02 % |
| 32 | CASTRO | 2 | 0 % |
| 33 | CHAMBERS | 98 | 0.11 % |
| 34 | CHEROKEE | 162 | 0.18 % |
| 35 | CHILDRESS | 1 | 0 % |
| 36 | CLAY | 85 | 0.1 % |
| 37 | COKE | 4 | 0 % |
| 38 | COLEMAN | 15 | 0.02 % |
| 39 | COLLIN | 3827 | 4.33 % |
| 40 | COLLINGSWORTH | 2 | 0 % |
| 41 | COLORADO | 20 | 0.02 % |
| 42 | COMAL | 1439 | 1.63 % |
| 43 | COMANCHE | 19 | 0.02 % |
| 44 | COOKE | 126 | 0.14 % |
| 45 | CORYELL | 35 | 0.04 % |
| 46 | CRANE | 3 | 0 % |
| 47 | CROSBY | 6 | 0.01 % |
| 48 | DALLAM | 2 | 0 % |
| 49 | DALLAS | 5153 | 5.83 % |
| 50 | DAWSON | 5 | 0.01 % |
| 51 | DEAF SMITH | 1 | 0 % |
| 52 | DENTON | 2421 | 2.74 % |
| 53 | DEWITT | 93 | 0.11 % |
| 54 | DICKENS | 1 | 0 % |
| 55 | DIMMIT | 13 | 0.01 % |
| 56 | DONLEY | 2 | 0 % |
| 57 | DUVAL | 7 | 0.01 % |
| 58 | EASTLAND | 10 | 0.01 % |
| 59 | ECTOR | 261 | 0.3 % |
| 60 | EDWARDS | 4 | 0 % |
| 61 | EL PASO | 811 | 0.92 % |
| 62 | ELLIS | 386 | 0.44 % |
| 63 | ERATH | 297 | 0.34 % |
| 64 | FALLS | 24 | 0.03 % |
| 65 | FANNIN | 33 | 0.04 % |
| 66 | FAYETTE | 76 | 0.09 % |
| 67 | FISHER | 6 | 0.01 % |
| 68 | FLOYD | 4 | 0 % |
| 69 | FORT BEND | 1776 | 2.01 % |
| 70 | FRANKLIN | 7 | 0.01 % |
| 71 | FREESTONE | 19 | 0.02 % |
| 72 | FRIO | 25 | 0.03 % |
| 73 | GAINES | 5 | 0.01 % |
| 74 | GALVESTON | 480 | 0.54 % |
| 75 | GARZA | 3 | 0 % |
| 76 | GILLESPIE | 143 | 0.16 % |
| 77 | GLASSCOCK | 1 | 0 % |
| 78 | GOLIAD | 81 | 0.09 % |
| 79 | GONZALES | 47 | 0.05 % |
| 80 | GRAY | 7 | 0.01 % |
| 81 | GRAYSON | 373 | 0.42 % |
| 82 | GREGG | 551 | 0.62 % |
| 83 | GRIMES | 58 | 0.07 % |
| 84 | GUADALUPE | 863 | 0.98 % |
| 85 | HALE | 26 | 0.03 % |
| 86 | HAMILTON | 23 | 0.03 % |
| 87 | HANSFORD | 1 | 0 % |
| 88 | HARDEMAN | 3 | 0 % |
| 89 | HARDIN | 250 | 0.28 % |
| 90 | HARRIS | 6271 | 7.09 % |
| 91 | HARRISON | 172 | 0.19 % |
| 92 | HARTLEY | 1 | 0 % |
| 93 | HASKELL | 2 | 0 % |
| 94 | HAYS | 1813 | 2.05 % |
| 95 | HEMPHILL | 8 | 0.01 % |
| 96 | HENDERSON | 194 | 0.22 % |
| 97 | HIDALGO | 527 | 0.6 % |
| 98 | HILL | 55 | 0.06 % |
| 99 | HOCKLEY | 35 | 0.04 % |
| 100 | HOOD | 99 | 0.11 % |
| 101 | HOPKINS | 19 | 0.02 % |
| 102 | HOUSTON | 36 | 0.04 % |
| 103 | HOWARD | 7 | 0.01 % |
| 104 | HUDSPETH | 3 | 0 % |
| 105 | HUNT | 107 | 0.12 % |
| 106 | Hutchinson | 5 | 0.01 % |
| 107 | IRION | 1 | 0 % |
| 108 | JACK | 10 | 0.01 % |
| 109 | JACKSON | 59 | 0.07 % |
| 110 | JASPER | 132 | 0.15 % |
| 111 | JEFF DAVIS | 1 | 0 % |
| 112 | JEFFERSON | 641 | 0.73 % |
| 113 | JIM WELLS | 55 | 0.06 % |
| 114 | JOHNSON | 295 | 0.33 % |
| 115 | JONES | 11 | 0.01 % |
| 116 | KARNES | 26 | 0.03 % |
| 117 | KAUFMAN | 233 | 0.26 % |
| 118 | KENDALL | 368 | 0.42 % |
| 119 | KENEDY | 1 | 0 % |
| 120 | KENT | 2 | 0 % |
| 121 | KERR | 400 | 0.45 % |
| 122 | KIMBLE | 9 | 0.01 % |
| 123 | KING | 1 | 0 % |
| 124 | KINNEY | 3 | 0 % |
| 125 | KLEBERG | 74 | 0.08 % |
| 126 | KNOX | 2 | 0 % |
| 127 | LA SALLE | 35 | 0.04 % |
| 128 | LAMAR | 69 | 0.08 % |
| 129 | LAMB | 10 | 0.01 % |
| 130 | LAMPASAS | 46 | 0.05 % |
| 131 | LAVACA | 96 | 0.11 % |
| 132 | LEE | 52 | 0.06 % |
| 133 | LEON | 26 | 0.03 % |
| 134 | LIBERTY | 43 | 0.05 % |
| 135 | LIMESTONE | 17 | 0.02 % |
| 136 | LIVE OAK | 11 | 0.01 % |
| 137 | LLANO | 190 | 0.21 % |
| 138 | LUBBOCK | 1586 | 1.79 % |
| 139 | LYNN | 18 | 0.02 % |
| 140 | MADISON | 20 | 0.02 % |
| 141 | MARION | 7 | 0.01 % |
| 142 | MARTIN | 12 | 0.01 % |
| 143 | MASON | 15 | 0.02 % |
| 144 | MATAGORDA | 19 | 0.02 % |
| 145 | MAVERICK | 9 | 0.01 % |
| 146 | MCCULLOCH | 2 | 0 % |
| 147 | MCLENNAN | 885 | 1 % |
| 148 | MEDINA | 169 | 0.19 % |
| 149 | MENARD | 5 | 0.01 % |
| 150 | MIDLAND | 508 | 0.57 % |
| 151 | MILAM | 41 | 0.05 % |
| 152 | MILLS | 3 | 0 % |
| 153 | MITCHELL | 2 | 0 % |
| 154 | MONTAGUE | 30 | 0.03 % |
| 155 | MONTGOMERY | 1906 | 2.16 % |
| 156 | MOORE | 4 | 0 % |
| 157 | MORRIS | 8 | 0.01 % |
| 158 | MOTLEY | 1 | 0 % |
| 159 | NACOGDOCHES | 322 | 0.36 % |
| 160 | NAVARRO | 84 | 0.1 % |
| 161 | NEWTON | 48 | 0.05 % |
| 162 | NOLAN | 19 | 0.02 % |
| 163 | NUECES | 1385 | 1.57 % |
| 164 | OLDHAM | 1 | 0 % |
| 165 | ORANGE | 224 | 0.25 % |
| 166 | PALO PINTO | 24 | 0.03 % |
| 167 | PANOLA | 24 | 0.03 % |
| 168 | PARKER | 247 | 0.28 % |
| 169 | PARMER | 4 | 0 % |
| 170 | POLK | 103 | 0.12 % |
| 171 | POTTER | 153 | 0.17 % |
| 172 | RAINS | 18 | 0.02 % |
| 173 | RANDALL | 318 | 0.36 % |
| 174 | Reagan | 2 | 0 % |
| 175 | REAL | 22 | 0.02 % |
| 176 | RED RIVER | 11 | 0.01 % |
| 177 | REEVES | 4 | 0 % |
| 178 | REFUGIO | 10 | 0.01 % |
| 179 | ROBERTS | 1 | 0 % |
| 180 | ROBERTSON | 79 | 0.09 % |
| 181 | ROCKWALL | 529 | 0.6 % |
| 182 | RUNNELS | 9 | 0.01 % |
| 183 | RUSK | 85 | 0.1 % |
| 184 | SABINE | 33 | 0.04 % |
| 185 | SAN AUGUSTINE | 18 | 0.02 % |
| 186 | SAN JACINTO | 35 | 0.04 % |
| 187 | SAN PATRICIO | 320 | 0.36 % |
| 188 | SAN SABA | 10 | 0.01 % |
| 189 | Scurry | 16 | 0.02 % |
| 190 | SHACKELFORD | 5 | 0.01 % |
| 191 | SHELBY | 23 | 0.03 % |
| 192 | SHERMAN | 3 | 0 % |
| 193 | SMITH | 2220 | 2.51 % |
| 194 | SOMERVELL | 17 | 0.02 % |
| 195 | STARR | 43 | 0.05 % |
| 196 | STEPHENS | 5 | 0.01 % |
| 197 | SWISHER | 7 | 0.01 % |
| 198 | TARRANT | 4232 | 4.79 % |
| 199 | TAYLOR | 326 | 0.37 % |
| 200 | TERRELL | 1 | 0 % |
| 201 | TERRY | 4 | 0 % |
| 202 | THROCKMORTON | 4 | 0 % |
| 203 | TITUS | 22 | 0.02 % |
| 204 | TOM GREEN | 85 | 0.1 % |
| 205 | TRAVIS | 16355 | 18.5 % |
| 206 | TRINITY | 23 | 0.03 % |
| 207 | TYLER | 29 | 0.03 % |
| 208 | UPSHUR | 140 | 0.16 % |
| 209 | UVALDE | 74 | 0.08 % |
| 210 | VAL VERDE | 8 | 0.01 % |
| 211 | VAN ZANDT | 89 | 0.1 % |
| 212 | VICTORIA | 1384 | 1.57 % |
| 213 | WALKER | 106 | 0.12 % |
| 214 | WALLER | 70 | 0.08 % |
| 215 | WARD | 3 | 0 % |
| 216 | WASHINGTON | 79 | 0.09 % |
| 217 | WEBB | 108 | 0.12 % |
| 218 | WHARTON | 43 | 0.05 % |
| 219 | WHEELER | 2 | 0 % |
| 220 | WICHITA | 1336 | 1.51 % |
| 221 | WILBARGER | 10 | 0.01 % |
| 222 | WILLACY | 9 | 0.01 % |
| 223 | WILLIAMSON | 7072 | 8 % |
| 224 | WILSON | 136 | 0.15 % |
| 225 | WISE | 57 | 0.06 % |
| 226 | WOOD | 83 | 0.09 % |
| 227 | YOUNG | 7 | 0.01 % |
| 228 | Zapata | 7 | 0.01 % |
| 229 | ZAVALA | 7 | 0.01 % |
| Grand Total | | **88406** | **100 %** |
| Note: There are 254 counties in Texas | | | |

Results
As mentioned in the discussion, the population sampled for antibody testing does not appear to be representative for the state as a whole (especially e.g. in gender [67% female] and ethnicity [90% white]). Would it therefore be useful to also adjust for these demographics in the calculations (which are just census-adjusted for age groups)?

We initially set out to adjust our TX Cares antibody positivity to the age and sex distribution of TX. However, we ultimately only adjusted for age in our analysis because 1.) the age distribution of vaccination differed markedly over time (due to differential rollout by age), and 2.) the age distribution of natural infection was also very different (Table 1). Thus, age adjustment was essential. While enrollment does differ substantially by sex, seropositivity does not differ by sex (Table 1: 27.2% for females an 26.5% for males as of Oct 31, 2021) so adjustment would not have altered these percentages.

An additional reason we could not adjust for both age and sex (statistically speaking, though we tried) is the following: the 5 strata of age groups (for which we must adjust), plus the 2 strata for sex created 10 total census strata (per each 1-week observation period so that a short-term period seroprevalence was obtained). Even with our large sample size, it was simply not sufficient to adequately draw inference on 10 total strata for a 1-week period (there were no people in some strata from our initial attempt to age- and sex- adjust). As such, we decided age was the most important adjustment factor, also due to the equal sex distribution in infection/antibody (27.2% and 26.5% in women and men, respectively). We now summarize this limitation, which would not affect the final total seropositivity estimates greatly, as the sex distribution is appx 50/50 in TX.

Finally, because the vaccinated population was state-derived aggregate data, it did not require adjustment as it was true and complete data. The Discussion now reads,

“We could not adjust for all demographic factors, e.g., sex, race, ethnicity, to the distribution in the Texas population. This is due to the fact that, for example, for 10 age strata and 2 sex strata one would have 20 strata over 1-week periods of estimation, often resulting in no participants in a given strata. Adding race and ethnicity adjustment, although ideal, would have further decreased strata size leading to unreliable statistical inference. While this is a limitation of the current study, it is notable that the natural antibody status was not different between men and women (26.5\% and 27.2\% respectively). There were race differences in antibody status, thus not standardizing remains a limitation of this study.”

Per the reviewer, we now also added additional demographic information on seropositivity to Table 1 (for the month of October 2021, to ensure period seroprevalence is being reported for that correct snapshot in time). This gives for a more nuanced demographic breakdown of positivity in our sample from 10/1/21 to 10/31/21:

New Table 1:

Demographics other than age (e.g. ethnicity) may also be particularly important in vaccine uptake rates. Were demographic data available for the vaccinated population?

We received all true Texas state vaccination data in aggregate form. Thus, we could not tease apart demographic effects as we did not have individual level data. However, we now link to the state Dashboard in our Introduction that shows granular data by county and demographics including ethnicity (data version Oct 31, 2021, and last accessed on July 12, 2022): <https://www.arcgis.com/apps/dashboards/45e18cba105c478697c76acbbf86a6bc>

Can Fig 1 & Fig 2 be combined as they contain the same data? This figure may also benefit from breaking down into demographic factors e.g. age and ethnicity to help identify target groups with high disease attack rates or high levels of susceptibility.

We answer the reviewer’s two sentences in 2 parts:

1. We decided to keep this as two figures for the following reasons: a.) The x axes are different. The Fig 1 x-axis starts on October 2020 when TX CARES began. The Fig 2 x-axis starts Jan 2021, when vaccination began. b.) Fig 1 shows the true estimated seropositivity (and 95% pointwise confidence band) from the prospective TX CARES cohort, since the start of the study in Oct 2020. Fig 2 shows an estimate using our modeling approach (after combining observed seropositivity and state-level vaccination).

However, if required by the reviewer or Editor, we can find a way to combine them.

2. Regarding breaking the plots into demographic factors such as age and ethnicity, please recall these plots are already directly standardized to the TX census for age, so we cannot then stratify by demographic factors such as age. Our new Table 1 partly alleviates this issue and responds to the reviewer’s point. The limitation is also now explained in the Discussion exactly as stated above,

“We could not adjust for all demographic factors, e.g., sex, race, ethnicity, to the distribution in the Texas population. This is due to the fact that, for example, for 10 age strata and 2 sex strata one would have 20 strata over 1-week periods of estimation, often resulting in no participants in a given strata. Adding race and ethnicity adjustment, although ideal, would have further decreased strata size leading to unreliable statistical inference. While this is a limitation of the current study, it is notable that the natural antibody status was not different between men and women (26.5\% and 27.2\% respectively). There were race differences in antibody status, thus not standardizing remains a limitation of this study.”

The abstract compared the estimated natural antibody rate of 35.2% with confirmed COVID-19 cases of 11.83%, but this was not mentioned in the paper itself. As the study included a large sample of 87,466, perhaps comparisons between seropositive and reported case reports may be possible for demographic-specific layers e.g. county, age, ethnicity, etc.

Thank you for drawing our attention to the oversight. Those percentages now appear in the Discussion for comparison purposes,

“The estimated natural antibody rate of 35.2% contrasts with state-confirmed COVID-19 cases of 11.83%, demonstrating the importance of population-level studies.”

Per the reviewer’s second sentence, please review the revised Table 1 as well as our 4 recently published papers that present demographic breakdowns of seropositivity of our longitudinal cohort at various time points in the study, as well as within individual over time (Messiah et al 2022a; Messiah et al 2022b, Valerio-Shewmaker et al 2021; Swartz et al 2022), which were all published while this manuscript was under review. We note that we submitted this manuscript before any of those published papers, but have ensured there is no overlapping data presentation.

Reviewer #2: Dear Editor,

I have now read the manuscript entitled: “Estimation of Natural and Vaccine-Induced Antibodies to SARS-CoV-2 in a Large Geographic Region” (manuscript no: PONE-D-21-40593) by DeSantis SM, Leon Novelo LG et al. The manuscript content exemplifies the result of using one SARS-CoV-2 serological assay (the Roche Elecsys called the Roche N-test) described to show neutralizing antibodies against the SARS-CoV-2 nucleocapsid (N) protein. Serum samples from a large population of 87 466 individuals (age span 0 – 85+years) from the state of Texas, USA were tested (study participants presented in Tables 1 and 2).
The study results seem interesting, but a number of issues in the manuscript need to be clarified and better explained.

Comments and questions:

It is interesting that the used serological assay is aimed at showing neutralizing antibodies against SARS-CoV-2 virus, and still the viral antigen seem to be the nucleoprotein of the virus. The nucleoprotein is an internal protein of the SARS-CoV-2 virus, and not exposed sufficiently on the surface of the infectious virus particle, so it is difficult to understand how the detected antibodies can be virus-neutralizing?

We use the Roche N test which tests for the nucleocapsid protein and not neutralizing antibodies. Since we submitted this manuscript, both antibodies have been shown to last as many as 6 months (including our own paper published in *JID*, Swartz et al 2022). You can see our revised Introduction including this new information and citations,

“Recent research suggests neutralizing and nucleocapsid antibodies to SARS-CoV-2 persist for at least 5-6 months~\citep{wajnberg2020robust,%Wajnberg et al, 2020; rodda2021functional,VanElslande,Swartz2022Antibody} or possibly longer \citep{deGiorgi}, and that re-infection risk is low in the several months after initial infection \citep{CDC:clincalcons}.

Citations:

1. Van Elslande J, Oyaert M, Ailliet S, Van Ranst M, Lorent N, Vande Weygaerde Y, André E, Lagrou K, Vandendriessche S, Vermeersch P. Longitudinal follow-up of IgG anti-nucleocapsid antibodies in SARS-CoV-2 infected patients up to eight months after infection. J Clin Virol. 2021 Mar;136:104765. doi: 10.1016/j.jcv.2021.104765. Epub 2021 Feb 18. PMID: 33636554; PMCID: PMC7891078.
2. Swartz MD, DeSantis SM, Yaseen A, Brito FA, Valerio-Shewmaker MA, Messiah SE, Leon-Novelo LG, Kohl HW, Pinzon-Gomez CL, Hao T, Zhang S, Talebi Y, Yoo J, Ross JR, Gonzalez MO, Wu L, Kelder SH, Silberman M, Tuzo S, Pont SJ, Shuford JA, Lakey D, Boerwinkle E.J Infect Dis. 2022 May 6:jiac167. doi: 10.1093/infdis/jiac167. Online ahead of print.

Q1. The authors should explain in which way the serological responses in the blood/serum samples of participating individuals can be virus-neutralizing if the assay target protein was an internal nucleocapsid virus protein?

Thank you for allowing us to clarify. We did not intend to state the detected antibodies from our Roche N test are virus- neutralizing, thus we removed the term, “virus-neutralizing” from our methods. The Roche nucleoprotein antibody test does not detect receptor binding domain antibodies specifically. We received guidance from Roche upon multiple meetings on how to interpret the positive result, which has 99.5% specificity.

We revised the Intro per above, as we ad other authors have shown virus neutralizing AND nucleoprotein antibodies may last at least 5-6 months. We now also add to methods,

“Based on Roche guidance, a positive result was assumed to be indication that natural infection had occurred.”

We also cite Swartz et al (2022) along with the previously cited Roche assay information in our citations.

Q2a. The vaccinated individuals in the study were vaccinated with the S1-spike presenting vaccines. Vaccines used were either mRNA-vaccines or Johnson and Johnson vaccine. The authors should reveal which mRNA vaccines were used (Please provide manufacturer and proportion of study participants that were given which of the vaccines).

Thank you for this important comment.

Per our revised Results, “As of Oct 31, 2021 55.9 \% (N=8.65 million) Texans received at least 2 doses Pfizer, 36.4\%(5.64 million) at least 2 doses of Moderna, and 7.7\% (1.19 million) at least one dose of Johnson and Johnson. Given this breakdown, our results largely reflect mRNA-induced antibodies” These are the state breakdowns, which are the (exact) data used in the current manuscript.

We note to the reviewer that this manuscript was submitted 10 months ago during the rapidly unfolding vaccine rollout. Appx 4-5 months later, this manuscript was passed to new Editors and reviewers (due to an Editorial departure). New knowledge about the 3 approved vaccines (and required # of boosters etc.) was acquired in this review time frame.

Q3. If the vaccine recipients in the study population were given S1-spike vaccine, and the used serological Roche-N-test contain only the nucleoprotein of SARS-CoV-2? How can the anti-S1-spike antibodies be made to react with the nucleoprotein if the nucleoprotein is not in the vaccine? Please clarify.

Please see above response as well as below.

This is not what was done. The Roche nucleoprotein test was applied to the TX CARES cohort and a positive result simply allowed us to assume prior infection. The vaccination data was true state level aggregate data. The two pieces of information were then combined using the statistical methodology presented.

As explained in the paper, we 1. Use true/correct vaccination data (2 doses of mRNA or 1 dose JNJ) to estimate those who would have vaccine-induced antibodies and 2. The TX CARES cohort Roche N test to count those who experienced SARS-CoV-2 infection. With the mathematics presented, we essentially add these percentages (% immune) together (census-adjusted for age) and “subtract out” the estimated overlap in percent immune (i.e., those who were vaccinated AND had natural infection), to ensure a reasonably unbiased estimate of “total immunity” from either vaccination or infection. The actual mathematical process is more complex than this statement (per the paper methodology), but that is what is accomplished.

Today, one can utilize an anti-Spike (S1) protein assay to ascertain who has any antibodies (vaccine or naturally induced). However, our statistical methodology is integral to pandemic preparedness since such a spike assay will generally not be available quickly in a novel pandemic. We have also changed the title of our manuscript to reflect the utility of the methodology by adding the word “Methodology” in front of the title. This puts slightly more focus on overall pandemic preparedness for mathematically estimating total immunity of a region.

Q4. In Materials and Methods, paragraph 2.1 the study populations are presented as students, or professionals, or children 5-17 years. However, in Table 1 also children between age 0-4 is given. What was the reason for including these young children? Where their serum also tested in the serological assays?

These 12 children 0-4 years did not enter the current analysis and have now been removed from Table 1. The total number of participants has changed by 12, which resulted in no change in the graph or total seropositivity given the large N of the study.

To explain the error: TX Cares was designed and approved for participants age 5+. There was an occasional participant from 0-4 ((N=12) whose parent entered their birthday incorrectly. All 12 children were reported to the institutional IRB and follow-up calls to parents ascertained their true age.

Q5a. Since the authors present their study populations in Tables 1 and 2 it would have been valuable to see how large proportions of the different groups that contained naturally infected respectively vaccinated individuals?.

We agree and did what you have asked for our TX Cares cohort. Please review the revised Table 1. We also refer you to our 4 relevant publications that were accepted during the review process of this manuscript (Messiah et al 2022a, Messiah et al 2022b, Swartz et al 2022, Valerio-Shewmaker 2021). As the vaccinate data are aggregate state-provided data we cannot show you these breakdowns but present the Texas State dashboard per above response.

Q5b) Similarly, which proportion received only the Johnson and Johnson vaccine once, and which proportion/category of study participant that received the mRNA vaccine twice?

Full vaccination was defined as 2 doses of an mRNA vaccine and 1 dose of Johnson & Johnson and we replied to this in Q2a. <https://www.arcgis.com/apps/dashboards/45e18cba105c478697c76acbbf86a6bc>

The proportion reporting full vaccination in our cohort by vaccine were: 29,519 (44.2%) Pfizer, 31,032 (46.49%) Moderna, 2,664 (3.99%) Johnson & Johnson, and 3,535 (5.39%) “I do not know/other.” These survey responses from our participants were not used since we had (true) state vaccination data, and given the possibility that people misunderstood the vaccine they received. However, they were used to determine the intersection of those reporting full vaccination and who had a positive N test, indicating the “overlap” described in our methodology.

Figures:
Q6. In figure 1 (Weekly natural immunity) Texas CARES Roche N-test), in the table-text …. “Seroprevalence by Age group” is claimed to be shown. However, this information look quite unclear in the graph provided?? The authors should explain how readers should be able to identify the seroprevalence for all age-groups studied (this information would be very valuable to see and would significantly enhance the interest of the obtained data in this manuscript !!).

We have modified the title of Fig 1 to “Weekly SARS-CoV-2 natural antibody period seroprevalence and pointwise 95% confidence band for Texas CARES, estimated with isotonic transformed and weighted seroprevalence, age-standardized to the Texas 2021 census.”

You are correct that we do not and cannot show seroprevalence by age group because we adjust for it using direct standardization to the 2021 census, which is CDC’s standard surveillance protocol.
